# Integrity and quantity of salivary cell‐free DNA as a potential molecular biomarker in oral cancer: A preliminary study

**DOI:** 10.1111/jop.13299

**Published:** 2022-04-26

**Authors:** Óscar Rapado‐González, José Luis López‐Cedrún, Ramón Manuel Lago‐Lestón, Alicia Abalo, Guillermo Rubin‐Roger, Ángel Salgado‐Barreira, Rafael López‐López, Laura Muinelo‐Romay, María Mercedes Suárez‐Cunqueiro

**Affiliations:** ^1^ Department of Surgery and Medical‐Surgical Specialties, Medicine and Dentistry School Universidade de Santiago de Compostela Santiago de Compostela Spain; ^2^ Liquid Biopsy Analysis Unit, Translational Medical Oncology Group (Oncomet) Health Research Institute of Santiago IDIS, Complexo Hospitalario Universitario de Santiago de Compostela (SERGAS) Santiago de Compostela Spain; ^3^ Centro de Investigación Biomédica en Red de Cáncer CIBERONC Instituto de Salud Carlos III Madrid Spain; ^4^ Department of Oral and Maxillofacial Surgery Complexo Hospitalario Universitario de A Coruña SERGAS A Coruña Spain; ^5^ Department of Preventive Medicine Universidade de Santiago de Compostela Santiago de Compostela Spain; ^6^ Epidemiology, Health Public and Health Technology Assessment, Health Research Institute of Santiago de Compostela (IDIS) Complexo Hospitalario Universitario de Santiago de Compostela (SERGAS) Santiago de Compostela Spain; ^7^ Translational Medical Oncology Group (Oncomet), Health Research Institute of Santiago (IDIS) Complexo Hospitalario Universitario de Santiago de Compostela (SERGAS) Santiago de Compostela Spain

**Keywords:** *ALU* repeat, DNA integrity, liquid biopsy, oral squamous cell carcinoma (OSCC), saliva, salivary cell‐free DNA (scfDNA)

## Abstract

**Background:**

Differences in cell‐free DNA (cfDNA) fragments have been described as a valuable tool to distinguish cancer patients from healthy individuals. We aim to investigate the concentration and integrity of cfDNA fragments in saliva from oral squamous cell carcinoma (OSCC) patients and healthy individuals in order to explore their value as diagnostic biomarkers.

**Methods:**

Saliva samples were collected from a total of 34 subjects (19 OSCC patients and 15 healthy controls). The total concentration of salivary cfDNA (scfDNA) was determined using a fluorometry method and quantitative real‐time polymerase chain reaction (qPCR). To evaluate the scfDNA quantity and integrity, qPCR targeting *Arthobacter luteus* (*ALU*) sequences at three amplicons of different lengths (60, 115, and 247 bp, respectively) was carried out. ScfDNA integrity indexes (*ALU*115/*ALU*60 and *ALU*247/*ALU*60) were calculated as the ratio between the absolute concentration of the longer amplicons 115 bp and 247 bp and the total scfDNA amount (amplicon 60 bp).

**Results:**

The total scfDNA concentration (*ALU*60) was higher in OSCC than in healthy donors, but this trend was not statistically significant. The medians of scfDNA integrity indexes, *ALU*115/*ALU*60 and *ALU*247/*ALU*60, were significantly higher in OSCC, showing area under the curve values of 0.8211 and 0.7018, respectively.

**Conclusion:**

Our preliminary results suggest that scfDNA integrity indexes (*ALU*115/*ALU*60 and *ALU*247/*ALU*60) have potential as noninvasive diagnostic biomarkers for OSCC.

## 1 INTRODUCTION

Oral squamous cell carcinoma (OSCC) is the most common head and neck malignancy, responsible for 377 713 new cases and 177 757 deaths worldwide in 2020.^1^ There is an urgent need to develop noninvasive biomolecular approaches for improving early detection and therapeutic success rates.^2^

Liquid biopsies have emerged as a potential noninvasive source of tumor‐related biomarkers.^3^ The analysis of cell‐free DNA (cfDNA) has aroused great interest as a noninvasive biomarker.^4^ The cfDNA comprises both nuclear and/or mitochondrial double‐stranded DNA released from the cells into the physiological body fluids, such as blood, saliva, urine, cerebrospinal fluid, or pleural fluid.^5^ The structural composition of cfDNA has been related to its origins including apoptosis, necrosis, or active release. Apoptotic cells secreted small and uniform DNA fragments, with a length around 167 bp as a result of caspase‐activated DNase (CAD) cleavage at internucleosomal chromatin regions. Conversely, necrotic cells release high‐molecular‐weight DNA due to random and incomplete genomic DNA fragmentation by DNases.^6^ The determination of cfDNA size distribution in plasma from cancer patients showed the presence of short and long DNA fragments derived from both apoptotic and necrotic cells,^7^ while plasma cfDNA from healthy individuals is only derived from apoptotic cells.^8^ Several studies have reported the presence of elevated levels of longer cfDNA fragments in serum, plasma, or urine from different types of tumors. Therefore, the DNA integrity index calculated as the ratio of the quantity of long to short DNA fragments has been described as a promising tumor biomarker.^9^, ^10^

Salivary cfDNA (scfDNA) has emerged as a potential biomarker in cancer^11^, ^12^; however, there is little information about its profile. Since saliva is the biological fluid of the oral cavity, scfDNA fragmentation could reflect a different origin under physiological and pathological conditions, representing a noninvasive biomarker for tumor detection. Our proposed hypothesis is that scfDNA fragments from OSCC patients are longer than those of healthy individuals due to tumor necrosis. Therefore, this study aimed to investigate whether the total scfDNA quantity and DNA integrity could be used for discriminating OSCC patients from healthy individuals.

## 2 MATERIALS AND METHODS

### 2.1 Study population

All primary OSCC patients were recruited at the Department of Oral and Maxillofacial Surgery from Complexo Hospitalario Universitario of A Coruña (CHUAC, SERGAS) in Galicia, Spain, between September 2020 and April 2021. All cases were confirmed by histopathological analysis of the corresponding tissue biopsy and classified based on human papillomavirus (HPV) status in negative or positive OSCC. HPV detection and genotyping were performed in tumor samples using the real‐time polymerase chain reaction (qPCR) assay Anyplex II HPV28 (Seegene, Seoul, South Korea). Anyplex II HPV28 allows to distinguish 28 HPV genotypes, including 13 high‐risk types (HPV‐16, ‐18, ‐31, ‐33, ‐35, ‐39, ‐45, ‐51, 52, ‐56, ‐58, ‐59, and ‐68), 9 low‐risk types (HPV‐6, ‐11, ‐40, ‐42, ‐43, ‐44, ‐53, ‐54, and ‐70), and 6 genotypes classified as probably carcinogenic (HPV‐26, ‐61, ‐66, ‐69, ‐73, and ‐82). None of the patients under study received surgery, chemotherapy, or radiotherapy before sample collection. Patients with the presence of synchronous tumor were excluded from the study. The staging of OSCC was established in accordance with the Eighth edition of the Tumor‐Node‐Metastasis (TNM)‐staging system promulgated by the American Joint Commission on Cancer and the International Union Against Cancer. Saliva samples were obtained before tissue biopsy, on the same day to avoid disease bias. The control group included healthy individuals without visible oral lesions and any acute or chronic inflammatory conditions. All participants provided written informed consent before enrollment. The present study was approved by the Galician Ethics Committee of Clinical Research (Ref. No. 2018/003) and carried out in accordance with principles outlined in the Declaration of Helsinki.

### 2.2 Saliva collection and DNA extraction

All the participants were asked to refrain from eating, drinking, smoking, and using oral hygiene products for at least 1 h prior to sample collection. Unstimulated saliva samples were collected using Danasaliva sample collection kit (Danagene, Ref. No 0603.43) according to the manufacturer's instructions. The saliva samples were taken to laboratory where they were stored at room temperature until processed (sample processing time less than 5 days). Then, saliva was centrifuged at 2600*g* for 15 min at room temperature. The salivary supernatant was removed from the pellet and centrifuged again at 13 000*g* for 1 min (two times) to avoid cell contamination. Salivary supernatants were stored at −80°C until further analysis.

DNA from salivary supernatants was extracted with the QIAamp DNA Circulating Nucleic Acid Kit (Qiagen, Ref. No. 55114), according to the manufacturer's instructions. DNA samples were stored at −20°C until use. Total DNA from each sample was quantified using the Qubit 4 Fluorometer (Thermo Fisher Scientific, Waltham, MA, USA). Then, Agilent's TapeStation 4200 (Agilent Technologies, Santa Clara, CA, USA) was used to assess the fragment distribution of the extracted salivary DNA through Agilent Genomic DNA Screen Tape assay (Agilent Technologies, Ref. No. 5067–5365), in order to get a visual approach of DNA integrity.

### 2.3 Bacterial scfDNA quantification assay

To determine the percentage of bacterial scfDNA, we carried out a qPCR‐based assay using microbial DNA qPCR Assay kit (Qiagen, Ref. No. 3520510), which is designed to detect bacterial *16S rRNA* gene. The reaction mixture for each direct qPCR contained 12.5 μL microbial qPCR mastermix, 1 μL microbial DNA qPCR Assay, and 11.5 μL of scfDNA in a total reaction volume of 25 μL. In addition, for the qPCR, a positive PCR control, a negative control, and a microbial DNA positive control were included following the manufacturer's protocol. A standard curve was performed with serial dilutions (20 ng/μL to 0.02 ng/μL) of ZymoBIOMICS™ microbial community DNA standard (Zymo, D6305). Thermal cycling was conducted on the QuantStudio™ 3 Real‐Time PCR system (Life Technologies, Foster City, CA, USA) using the following parameters: initial denaturation at 95°C for 10 min followed by 40 cycles of amplification at 95°C for 15 s and 60°C for 2 min.

### 2.4 qPCR of *ALU* fragments

The quantity of the cfDNA in saliva was evaluated by qPCR targeting the human *Arthobacter luteus* (*ALU*) gene. The length of the amplicons designed for this study was 60, 115, and 247 bp, respectively. The sequences of *ALU*60 primers were forward: 5′‐ACCAGCCTGGCCAAC‐3′ and reverse 5′‐GCCCGGCTAATTTTTGTA‐3′; *ALU*115 primers were forward: 5′‐CCTGAGGTCAGGAGTTCGAG‐3′ and reverse: 5′‐CCCGAGTAGCTGGGATTACA‐3′; and *ALU*247 primers were forward: 5′‐GTGGCTCACGCCTGTAATC‐3′ and reverse: 5′‐CAGGCTGGAGTGCAGTGG‐3′. The primer set for *ALU*60 amplified both short (apoptotic) and long (nonapoptotic) DNA fragments representing the total amount of scfDNA.

The reaction mixture for each direct qPCR contained 10 μl TaqMan™ Gene Expression Master Mix (ThermoFischer), 4 μl H_2_0, 1 μl of each primer, and 5 μl of DNA in a total reaction volume of 20 μl. For each unknown reaction, 30 ng of DNA extract was added and for the negative control reaction, 5 μl of DNAse‐free water was added. Thermal cycling setting was performed in order to achieve a comparable qPCR efficiency among the three different amplicons. The qPCR protocol included an initial denaturation step at 95°C for 10 min and 50 cycles of PCR as follows: (i) 15 s at 95°C, 60 s at 60°C, and 60 s at 72°C for the for 60 bps amplicon; (ii) 15 s at 95°C, 60 s at 60°C, and 60 s at 72°C for the longer amplicons for 115 and 247 bp amplicons using QuantStudio™ 3 Real‐Time PCR system. The absolute equivalent amount of DNA fragments in each sample was determined by a standard curve with serial dilutions (20 ng to 2 pg) of prepared human genomic DNA (Sigma‐Aldrich, 116911120001). All the measurements were performed in duplicate.

### 2.5 scfDNA integrity index

Each integrity index was calculated as the ratio between the absolute concentration of the longer amplicons (amplicon 115 and 247 bp) and the total scfDNA amount (amplicon 60 bp). Then, scfDNA integrity was considered as *ALU*115/*ALU*60 and *ALU*247/*ALU*60. In addition, we evaluated the fraction of scfDNA fragments with lengths varying from 60 to 115 bp and from 115 to 247 bp by subtracting the absolute concentration of the longer fragment to that of the shorter one. Also, longer scfDNA fragments (≥247 bp) were calculated. These results were normalized on the total scfDNA amount and expressed as percentages.

### 2.6 Statistical analysis

The sample size was calculated using G*Power version 3.1.9.7 according to the median and interquartile range of DNA integrity index reported by Azab et al. for OSCC patients and healthy control group.^13^ The sample size was calculated with an effect size of 1.055, a level of significance of 0.05, and a power of 80%. Statistical analyses were performed with IBM SPSS Statistics 20, and graphs were generated using GraphPad Prism 5.0 (GraphPad Software, Inc., San Diego, CA, USA). Continuous variables were expressed as medians, interquartile range (IQR 25–75), and categorical variables were expressed as counts and percentages. Statistical differences between quantitative data were evaluated by a two‐tailed Mann–Whitney *U* test while the relationship between continuous with categorical variables was investigated using the Chi‐square test or, where appropriate, Fisher's exact test. Receiver operating characteristic (ROC) curves were constructed, and area under the ROC curve (area under the curve [AUC]) with 95% of confidence intervals (CIs) was obtained to evaluate the diagnostic accuracy of using scfDNA integrity index for OSCC. The cut‐off selection was made based on the value that provided the highest sensitivity and specificity to discriminate OSCC patients versus healthy controls. *p*‐value <0.05 was set as the level of statistical significance.

## 3 RESULTS

### 3.1 Demographic and clinical characteristics of enrolled participants

A total of 34 subjects, 19 OSCC patients (11 male and 8 female) with a median (IQR) age of 72 (67–76) and 15 healthy controls (7 male and 8 female), with a median age of 68 (64–75) were included in this study. Selected subjects did not show statistically differences by age (*p* = 0.5085) and gender (*p* = 0.730). Clinicopathological characteristics of OSCC patients are shown in Table 1. According to TNM stage, 12 patients were at Stages I and II and 7 at Stages III and IV. Moreover, the histological grade was well/moderate in 18 OSCC patients.

**TABLE 1:** Clinicopathological characteristics of study subjects

	OCSCC, *N* = 19	Healthy individuals, *N* = 15
Age, years		
Median (IQR 25–75)	72 (67–76)	68 (64–75)
Gender, *n* (%)		
Male	11 (57.9)	7 (46.7)
Female	8 (42.1)	8 (53.3)
Smoking status, *n* (%)		
Current	4 (21.05)	
Never	15 (78.9)	
Daily alcohol consumption		
Yes	2 (10.5)	
No	17 (89.5)	
OPMD, *n* (%)		
Yes	5 (26.3)	
No	14 (73.7)	
Tumor location		
Tongue	12 (63.2)	
Floor of mouth	5 (26.3)	
Gingiva	2 (10.5)	
Tumor stage (TNM)		
Early stages (I and II)	12 (63.2)	
Late stages (III and IV)	7 (36.8)	
Histologic grade		
Well	2 (10.5)	
Moderate	16 (84.2)	
Poor	1 (5.3)	
Tumor HPV status, *n* (%)		
HPV positive	2 (10.5)	
HPV negative	17 (89.5)	

Abbreviations: HPV, human papillomavirus; IQR, interquartile range; OPMD, oral potentially malignant disorders; TNM, Tumor‐Node‐Metastasis.

### 3.2 scfDNA quantitation and integrity

The median (IQR) concentration of scfDNA obtained in OSCC was 6.200 ng/ml (2.500–11.233) whereas in healthy individuals was 4.333 ng/ml (1.080–14.467) by a fluorometric method (Qubit). Although scfDNA concentration was increased in cancer patients, no significant differences were observed (*p* = 0.3492). In addition, we analyzed the potential contamination of bacterial‐derived DNA in the samples. Thus, after applying the qPCR assay to detect bacterial DNA in 19 samples from OSCC patients, the median (IQR) concentration was 71.67 (26.28–145.8) ng/ml. The mean percentage of bacterial DNA, as we expected, was negligible in comparison with the human DNA since only 3.5% of the total scfDNA quantified in OSCC patients was associated with bacterial DNA.

The qPCR assay was used to determine more specifically the concentration of short and long scfDNA fragments. The median (IQR) *ALU*60 concentration in OSCC patients and healthy controls was 6.404 ng/ml (2.721–9.748) and 2.715 ng/ml (1.997–19.938) ng/ml, respectively (*p* = 0.5324). The median value of *ALU*115 amplicon was again higher in OSCC patients (3.524 ng/ml; IQR: 1.439–6.150) than in healthy controls (1470 ng/ml; IQR: 6.753–7.426) ng/ml (*p* = 0.3190). Finally, the median of *ALU*247 in OSCC patients and healthy controls was 1.907 (838.5–4.898) and 1.205 (477.3–3.270) ng/ml, respectively (*p* = 0.3190). Overall, the results show a decrease in scfDNA concentration as the amplicon dimension increase both in OSCC patients and healthy controls.

### 3.3 scfDNA integrity indexes by qPCR

**FIGURE 1:**
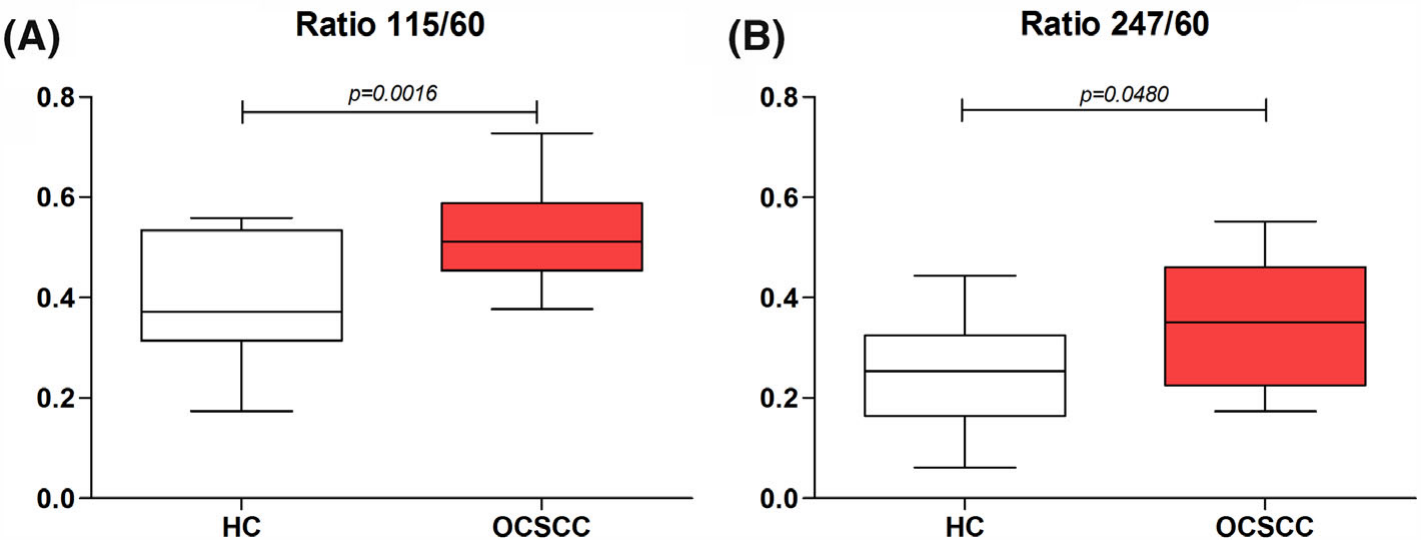
ScfDNA integrity indexes in OCSCC patients and HC: (A) Ratio *ALU115/ALU60* and (B) Ratio *ALU247/ALU60*. HC, healthy controls; OCSCC, oral cavity squamous cell carcinoma; scfDNA, salivary cell‐free DNA

The median salivary DNA integrity *ALU*115/*ALU*60 was significantly higher in OSCC patients (0.5120; IQR: 0.4540–0.5886) compared to healthy individuals (0.3725; IQR: 0.3141–0.5342) (*p* = 0.0016) (Figure 1A). Similarly, the median salivary DNA integrity *ALU*247/*ALU*60 was significantly higher in OSCC patients (0.3503; IQR: 0.2248–0.4612) compared to healthy individuals (0.2531; IQR: 0.1640–0.3247) (*p* = 0.048) (Figure 1B).

**FIGURE 2:**
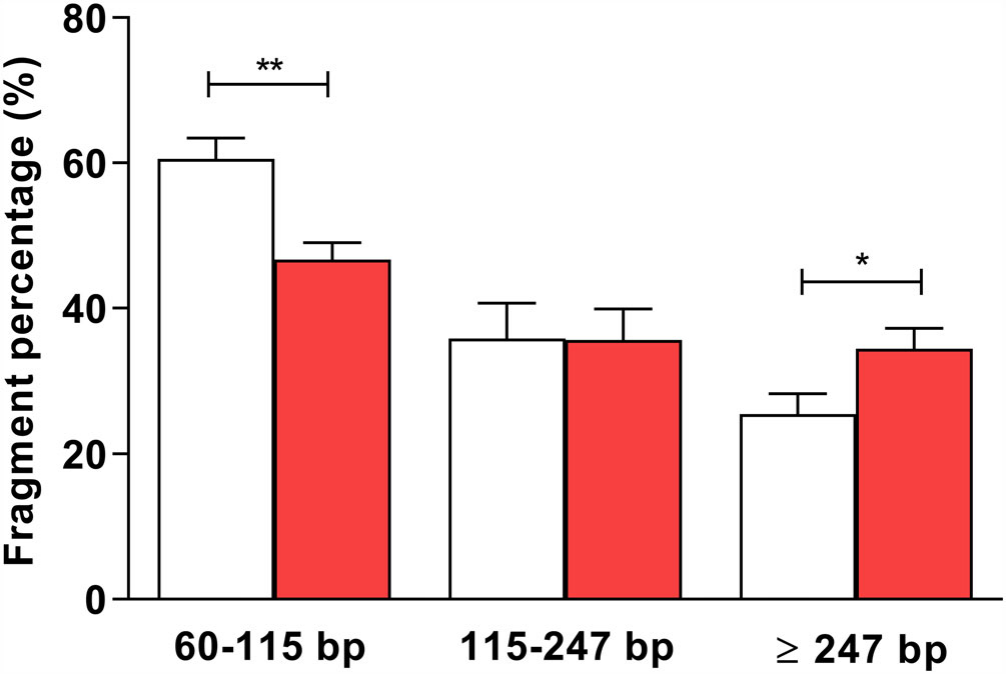
Percentages of scfDNA fragments of different length ranging 60–115, 115–247, and ≥247 in OCSCC patients (red column) and HC (white columm). *p* < 0.05; **p < 0.01. HC, healthy controls; OCSCC, oral cavity squamous cell carcinoma; scfDNA, salivary cell‐free DNA

Analyzing the amount of scfDNA fragments by qPCR (Figure 2), we observed that 62% of the total scfDNA in healthy controls comprised fragments with lengths ranging from 60 to 115 bp. However, this percentage was significantly lower in OSCC patients (*p* = 0.0016). Regarding the percentage of fragments comprised between 115 and 247 bp, no significant differences were observed between OSCC patients and healthy individuals, representing around 35% of total scfDNA fragments (*p* = 0.8642). On the contrary, the percentage of longer fragments (≥247 bp) with respect to total scfDNA quantity was significantly higher in OSCC patients (35%) than in healthy controls (25%) (*p* = 0.048).

### 3.4 Diagnostic utility of scfDNA integrity indexes

**FIGURE 3:**
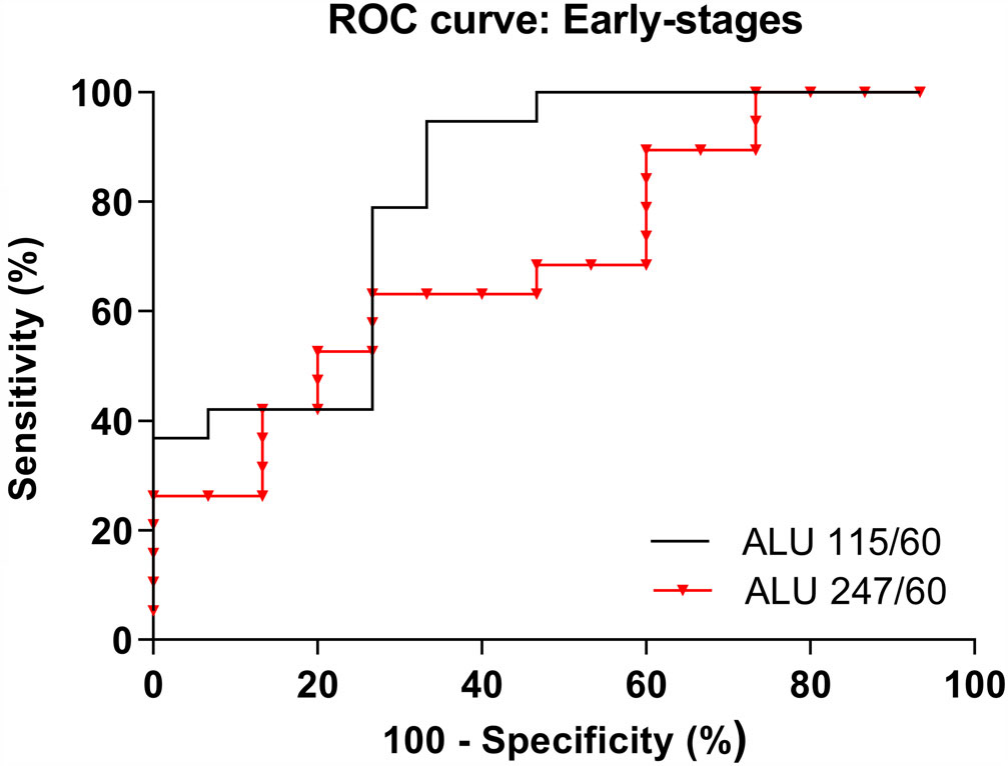
ROC curves of scfDNA integrity indexes *ALU115/ALU60* and *ALU247/ALU60* for discriminating OCSCC patients from HC. HC, healthy controls; OCSCC, oral cavity squamous cell carcinoma; ROC, receiver operating characteristic; scfDNA, salivary cell‐free DNA

The ROC curves for discriminating OSCC patients from healthy individuals showed AUC values of 0.8211 (95% CI, 0.6722–0.9699) for scfDNA integrity index *ALU*115/*ALU*60 and 0.7018 (95% CI, 0.5253–0.8782) for scfDNA integrity index *ALU*247/*ALU*60 (Figure 3). At the optimal cut‐off level of >0.4479 ng/ml, the scfDNA integrity index *ALU*115/*ALU*60 showed 83.33% sensitivity and 73.33% specificity. The scfDNA integrity index *ALU*247/*ALU*60 also yielded 83.33% sensitivity and 73.33% specificity at the cut‐off level of >0.2661 ng/ml.

## 4 DISCUSSION

Liquid biopsies based on tumor biomarkers represent an opportunity for improving early cancer detection. Among the various liquid biopsy samples, saliva has attracted great interest in identifying tumor‐related biomarkers since its collection is easy, noninvasive, and inexpensive.^14^ Like in blood, in addition to the cellular fraction, saliva comprises an extracellular fraction that reflects local and systemic biological information.^15^, ^16^ As we previously noted, scfDNA could be released by apoptosis, necrosis, or active secretion from malignant and nonmalignant cells.^12^ Recently, evidence has demonstrated the feasibility to detect tumor‐derived circulating DNA in saliva.^11^ Nevertheless, the potential clinical role of scfDNA concentration and integrity for OSCC diagnosis was not already established.

To the best of our knowledge, this is the first study that investigates the potential application of cfDNA concentration and cfDNA integrity using saliva for discriminating OSCC patients from healthy controls. The cfDNA concentration has been shown to act as a diagnostic biomarker due to its ability to differentiate cancer patients from controls subjects.^17^, ^18^ Opposite to other tumor types, few studies analyzed the quantity of cfDNA in oral cancer.^19^, ^20^, ^21^ In this preliminary study, we have observed using fluorometry and *ALU*60‐qPCR that scfDNA levels were higher in OSCC patients compared to healthy controls, but this increase was not statistically significant, probably because of the limited cohort analyzed. Similarly, Ding et al. analyzed the concentration of scfDNA in lung cancer by LINE1‐qPCR, but no significant differences were observed among the groups.^22^ Previously, Shukla et al. failed to find significant differences in plasma cfDNA levels in oral cancer patients, oral potentially malignant disorders, or patients with oral cancer after surgery compared with healthy controls.^20^ By contrary, Lin et al. reported a significant increase of cfDNA levels in oral cancer, showing an elevation of plasma cfDNA concentration with tumor progression.^19^ Based on our findings, the total concentration of scfDNA may have limited diagnostic value for oral cancer detection and not provides information about the mechanism of releasing cfDNA into saliva.

In addition to the total scfDNA concentration, the scfDNA fragment size may be used to differentiate cancer patients from healthy controls. In the last few years, several studies have focused on the analysis of cfDNA integrity as a promising tumor biomarker.^9^, ^23^, ^24^ Since apoptosis is the main source of uniform and small fragments of circulating cfDNA in healthy individuals, the presence of longer cfDNA fragments has been referred to as a marker of necrotic tumor cell death. Therefore, the ratio of longer to shorter fragments, known as cfDNA integrity, reflects the relative amount of nonapoptotic cell death.^9^ *ALU*‐targeting qPCR represents the most common method for assessing DNA integrity, specifically *ALU*115 and *ALU*247 are the sequences analyzed to determine DNA originating from apoptotic and necrotic cell death, respectively.^9^, ^10^, ^25^, ^26^ Like Ren et al.,^27^ we have used three sets of *ALU* primers: two primer sets for the amplicons with 60 and 115 bp lengths that amplify both short and long DNA fragments, and a primer set for the amplicon 247 bp that amplifies only longer DNA fragments. *ALU* sequences are transposable DNA elements (short interspersed elements) of around 300 bp length that comprise more than 10% of the mass of the human genome.^28^ In our study, we have observed that the medians for the two scfDNA integrity indexes (*ALU*115/*ALU*60 and *ALU*247/*ALU*60) were significantly higher in OSCC patients compared to healthy controls. In line with our results, Desai et al.^21^ showed in plasma from oral cancer patients a significantly higher DNA integrity compared to healthy or oral potentially malignant disorders groups, using the ratio of longer (253 bp) to shorter (102 bp) beta‐actin gene fragments. Furthermore, considering the diagnostic potential of each scfDNA integrity index, we observed that the scfDNA integrity index 115/60 had the highest diagnostic accuracy (AUC = 0.8211) for discriminating OSCC with a sensitivity and specificity of 83.33% and 73.33%, respectively. In line with our findings, Pinzani et al.^23^ reported by the quantification of four amplicons length of the Amyloid Precursor Protein gene that the ratio 180/67 had the better diagnostic accuracy for differentiating melanoma patients compared to the ratios 307/67 and 406/67. Recently, the study performed by Azab et al.^13^ based on cfDNA from oral rinses found a poor diagnostic accuracy of DNA integrity index *ALU*247/*ALU*115 for discriminating oral cancer from oral potentially malignant disorders (leukoplakia and oral lichen planus), showing an AUC of 0.652. In addition, our results regarding the scfDNA fragment size revealed that longer scfDNA fragments (>247 bp) are significantly more prevalent in OSCC while in healthy controls there is a prevalence of shorter fragments between 60 and 115 bp, suggesting tumor necrosis would be increasing the relative concentration of longer DNA fragments in OSCC patients. Therefore, these results indicate that OSCC patients have scfDNA with higher integrity, probably for the presence of a higher content of necrotic DNA and this integrity represents a potential diagnosis tool that should be further explored in this type of tumor. Interestingly, our study revealed for the first time also the presence of scfDNA of high molecular weight in healthy individuals indicating that nontumoral scfDNA is innately a mixture of longer and shorter DNA fragments. We hypothesized that the global profile of scfDNA could be conditioned by the content of salivary exosomes and the mechanisms of epithelial cell death modulated by the oral microbiota.^29^, ^30^

However, our study is not exempt from shortcomings. Our findings should be validated in longitudinal studies with larger sample sizes and including a control group of oral potentially malignant disorders for a better understanding of the potential clinical impact of scfDNA, not only for diagnosis but also for prognosis and monitoring the response to therapy in OSCC. Also, future studies should investigate the correlation between cfDNA integrity in saliva and plasma to determine the effectiveness of each biofluid in early‐ and late‐stage cancer. To optimize the diagnostic accuracy, the combination of scfDNA integrity with other exploratory tumor‐specific biomarkers such as proteins, microRNAs, or other biomolecules should be explored.

In conclusion, scfDNA integrity indexes (*ALU*115/60 and *ALU*247/60) could be promising biomarkers for discriminating OSCC patients from healthy controls. Our results, although representing a pilot study, open new avenues to explore scfDNA as a clinical tool.

## CONFLICT OF INTEREST

R. López‐López reports other from Nasasbiotech, during the conduct of the study; grants and personal fees from Roche, grants and personal fees from Merck, personal fees from AstraZeneca, personal fees from Bayer, personal fees, and nonfinancial support from BMS, personal fees from Pharmamar, personal fees from Leo, outside the submitted work. The rest of the authors have nothing to disclose.

## AUTHOR CONTRIBUTIONS

**O. Rapado‐González:** conceptualization, data curation, formal analysis, investigation, visualization, writing—original draft, and writing—review and editing. **J. L. López‐Cedrún:** data curation and visualization. **R. M. Lago‐Lestón:** methodology. **A. Abalo:** data curation. **G. Rubin‐Roger:** data curation. **A. Salgado‐Barreira:** methodology; **R. López‐López:** funding acquisition and supervision; **L. Muinelo‐Romay:** conceptualization, investigation, writing—review and editing; **M. M. Suárez‐Cunqueiro:** conceptualization, funding acquisition, investigation, project administration, supervision, writing—original draft, writing—review and editing.

## Data Availability

The data that support the findings of this study are available on request from the corresponding author. The data are not publicly available due to privacy or ethical restrictions.

## References

[1] Sung H , Ferlay J , Siegel RL , et al. Global Cancer Statistics 2020: GLOBOCAN estimates of incidence and mortality worldwide for 36 cancers in 185 countries. CA Cancer J Clin. 2021;71:209‐249. 33538338 10.3322/caac.21660

[2] Lousada‐Fernandez F , Rapado‐Gonzalez O , Lopez‐Cedrun J‐L , Lopez‐Lopez R , Muinelo‐Romay L , Suarez‐Cunqueiro M . Liquid biopsy in oral cancer. Int J Mol Sci. 2018;19:1704. 10.3390/ijms19061704 PMC6032225 29890622

[3] Siravegna G , Marsoni S , Siena S , Bardelli A . Integrating liquid biopsies into the management of cancer. Nat Rev Clin Oncol. 2017;14:531‐548. 28252003 10.1038/nrclinonc.2017.14

[4] Wan JCM , Massie C , Garcia‐Corbacho J , et al. Liquid biopsies come of age: towards implementation of circulating tumour DNA. Nat Rev Cancer. 2017;17:223‐238. 28233803 10.1038/nrc.2017.7

[5] Thierry AR , El Messaoudi S , Gahan PB , Anker P , Stroun M . Origins, structures, and functions of circulating DNA in oncology. Cancer Metastasis Rev. 2016;35:347‐376. 27392603 10.1007/s10555-016-9629-x PMC5035665

[6] Heitzer E , Auinger L , Speicher MR . Cell‐free DNA and apoptosis: how dead cells inform about the living. Trends Mol Med. 2020;26:519‐528. 32359482 10.1016/j.molmed.2020.01.012

[7] Jahr S , Hentze H , Englisch S , et al. DNA fragments in the blood plasma of cancer patients: quantitations and evidence for their origin from apoptotic and necrotic cells. Cancer Res. 2001;61:1659‐1665. 11245480

[8] Suzuki N , Kamataki A , Yamaki J , Homma Y . Characterization of circulating DNA in healthy human plasma. Clin Chim Acta. 2008;387:55‐58. 17916343 10.1016/j.cca.2007.09.001

[9] Umetani N , Giuliano AE , Hiramatsu SH , et al. Prediction of breast tumor progression by integrity of free circulating DNA in serum. J Clin Oncol. 2006;24:4270‐4276. 16963729 10.1200/JCO.2006.05.9493

[10] Hussein NA , Mohamed SN , Ahmed MA . Plasma ALU‐247, ALU‐115, and cfDNA integrity as diagnostic and prognostic biomarkers for breast cancer. Appl Biochem Biotechnol. 2019;187:1028‐1045. 30151636 10.1007/s12010-018-2858-4

[11] Li F , Wei F , Huang WL , et al. Ultra‐short circulating tumor DNA (usctDNA) in plasma and saliva of non‐small cell lung cancer (NSCLC) patients. Cancers (Basel). 2020;12:2041. 10.3390/cancers12082041 PMC7464208 32722209

[12] Rapado‐González Ó , Muinelo‐Romay L , Suárez‐Cunqueiro MM . Letter to the editor: “Liquid biopsy based on saliva cell‐free DNA as a potential biomarker for head and neck cancer”. Oral Oncol. 2021;112:105016. 32972862 10.1016/j.oraloncology.2020.105016

[13] Azab N , Zahran F , Amin A , Rady N . DNA integrity in diagnosis of premalignant lesions. Med Oral Patol Oral Cir Bucal. 2021;26:e445‐e450. 33340077 10.4317/medoral.24287 PMC8254884

[14] Kaczor‐Urbanowicz KE , Wei F , Rao SL , et al. Clinical validity of saliva and novel technology for cancer detection. Biochim Biophys Acta Rev Cancer. 2019;1872:49‐59. 31152821 10.1016/j.bbcan.2019.05.007 PMC6692231

[15] Elashoff D , Zhou H , Reiss J , et al. Prevalidation of salivary biomarkers for oral cancer detection. Cancer Epidemiol Biomarkers Prev. 2012;21:664‐672. 22301830 10.1158/1055-9965.EPI-11-1093 PMC3319329

[16] Rapado‐González Ó , Majem B , Álvarez‐Castro A , et al. A novel saliva‐based miRNA signature for colorectal cancer diagnosis. J Clin Med. 2019;8:2029. 10.3390/jcm8122029 PMC6947363 31757017

[17] Brisuda A , Pazourkova E , Soukup V , et al. Urinary cell‐free DNA quantification as non‐invasive biomarker in patients with bladder cancer. Urol Int. 2015;96:25‐31. 26338254 10.1159/000438828

[18] Yoon KA , Park S , Lee SH , Kim JH , Lee JS . Comparison of circulating plasma DNA levels between lung cancer patients and healthy controls. J Mol Diagn. 2009;11:182‐185. 19324991 10.2353/jmoldx.2009.080098 PMC2671334

[19] Lin LH , Chang KW , Kao SY , Cheng HW , Liu CJ . Increased plasma circulating cell‐free DNA could be a potential marker for oral cancer. Int J Mol Sci. 2018;19:3303. 10.3390/ijms19113303 PMC6274798 30352977

[20] Shukla D , Kale AD , Hallikerimath S , Yerramalla V , Subbiah V . Can quantifying free‐circulating DNA be a diagnostic and prognostic marker in oral epithelial dysplasia and oral squamous cell carcinoma? J Oral Maxillofac Surg. 2013;71:414‐418. 22749518 10.1016/j.joms.2012.04.039

[21] Desai A , Kallianpur S , Mani A , et al. Quantification of circulating plasma cell free DNA fragments in patients with oral cancer and precancer. Gulf J Oncolog. 2018;1:11‐17. 30145546

[22] Ding S , Song X , Geng X , et al. Saliva‐derived cfDNA is applicable for EGFR mutation detection but not for quantitation analysis in non‐small cell lung cancer. 2019;10:1973‐1983. 10.1111/1759-7714.13178 PMC6775000 31441578

[23] Pinzani P , Salvianti F , Zaccara S , et al. Circulating cell‐free DNA in plasma of melanoma patients: qualitative and quantitative considerations. Clin Chim Acta. 2011;412:2141‐2145. 21839068 10.1016/j.cca.2011.07.027

[24] Vizza E , Corrado G , De Angeli M , et al. Serum DNA integrity index as a potential molecular biomarker in endometrial cancer. J Exp Clin Cancer Res. 2018;37:16. 29382392 10.1186/s13046-018-0688-4 PMC5791183

[25] Soliman SES , Alhanafy AM , Habib MSED , Hagag M , Ibrahem RAL . Serum circulating cell free DNA as potential diagnostic and prognostic biomarker in non small cell lung cancer. Biochem Biophys Rep. 2018;15:45‐51. 29984326 10.1016/j.bbrep.2018.06.002 PMC6031238

[26] Feng J , Gang F , Li X , et al. Plasma cell‐free DNA and its DNA integrity as biomarker to distinguish prostate cancer from benign prostatic hyperplasia in patients with increased serum prostate‐specific antigen. Int Urol Nephrol. 2013;45:1023‐1028. 23779229 10.1007/s11255-013-0491-2

[27] Ren S , Ren X , Guo H , et al. Concentration and integrity indexes of urine cell‐free DNA as promising biomarkers for early lung cancer diagnosis. Per Med. 2021;18:129‐139. 33565322 10.2217/pme-2020-0019

[28] Batzer MA , Deininger PL . Alu repeats and human genomic diversity. Nat Rev Genet. 2002;3:370‐379. 11988762 10.1038/nrg798

[29] White T , Alimova Y , Alves VTE , et al. Oral commensal bacteria differentially modulate epithelial cell death. Arch Oral Biol. 2020;120:104926. 33096404 10.1016/j.archoralbio.2020.104926 PMC7655725

[30] Malkin EZ , Bratman SV . Bioactive DNA from extracellular vesicles and particles. Cell Death Dis. 2020;11:584. 32719324 10.1038/s41419-020-02803-4 PMC7385258

